# Pattern of disease and determinants of mortality among ICU patients on mechanical ventilator in Sub-Saharan Africa: a multilevel analysis

**DOI:** 10.1186/s13054-023-04316-w

**Published:** 2023-01-24

**Authors:** Semagn Mekonnen Abate, Bivash Basu, Bedru Jemal, Siraj Ahmed, Bahru Mantefardo, Tagesse Taye

**Affiliations:** 1 Department of Anesthesiology, College of Health Sciences and Medicine, Dilla University, Dilla, Ethiopia; 2 Departemnt of Internal Medicine, College of Health Sciences and Medicine, Dilla University, Dilla, Ethiopia; 3 Department of Anesthesiology, College of Health Sciences and Medicine, Hawassa University, Hawassa, Ethiopia

## Abstract

**Background:**

The global mortality rate of patients with MV is very high, despite a significant variation worldwide. Previous studies conducted in Sub-Saharan Africa among ICU patients focused on the pattern of admission and the incidence of mortality. However, the body of evidence on the clinical outcomes among patients with MV is still uncertain.

**Objective:**

The objective of this study was to investigate the pattern of disease and determinants of mortality among patients receiving mechanical ventilation in Southern Ethiopia.

**Methods:**

Six hundred and thirty patients on mechanical ventilation were followed for 28 days, and multilevel analysis was used to account for the clustering effect of ICU care in the region.

**Results:**

The incidence of 28-day mortality among patients with MV was 49% (95% CI: 36–58). The multilevel multivariate analysis revealed that being diabetic, having GSC < 8, and night time admission (AOR = 7.4; 95% CI: 2.96–18.38), (AOR = 5.9; (5% CI: 3.23, 10.69), and (AOR = 2.5; 95% CI: 1.24, 5.05) were predictors.

**Conclusion:**

The higher 28-day mortality among ICU patients on mechanical ventilation in our study might be attributed to factors such as delayed patient presentation, lack of resources, insufficient healthcare infrastructure, lack of trained staff, and financial constraints.

*Trial Registration*. The protocol was registered retrospectively on (NCT05303831).

**Supplementary Information:**

The online version contains supplementary material available at 10.1186/s13054-023-04316-w.

## Background

The burden of life-threatening conditions requiring mechanical ventilation (MV) in the intensive care unit has grown substantially in the last couple of decades in low- and middle-income countries because of an emerging pandemic, motorization, urbanization, and hospital expansion [1–6]. However, the advancement of ICU care is very limited due to the high cost of infrastructure, training, medical staff, failure to incorporate international guidelines for evidence-based care, and inadequate medical supplies [3, 5, 7–10].

Epidemiological studies revealed that as high as 70% of patients admitted to the intensive care unit (ICU) require mechanical ventilation at some point during their stay in ICU [11, 12].

An international prospective cohort study of 15,757 patients admitted to the ICU found that 5183 (33%) were on mechanical ventilation for more than 12 h, with a third of them suffering from ARDS [12]. Another multicenter study conducted in Poland by Kubler et al. among eighty-three ICUs across the country found that more than seventy percent of patients were on mechanical ventilation with respiratory failure (40%), coma (40%), chronic obstructive pulmonary disease (14%), and neuromuscular disease (5%) [11].

Studies investigating the incidence of MV and determinants of mortality among mechanically ventilated patients in Sub-Saharan African countries are very limited. A few studies in Ethiopia revealed that patients requiring mechanical ventilation and the indication for mechanical ventilation greatly vary from region to region [13–20].

Evidence revealed that the outcomes of patients with MV varied widely from one population to another, which is greatly influenced by ICU infrastructure, patient characteristics, emergent clinical events, ICU staff profile, integrated patient monitors, and medical supplies [11, 21–23].

Studies on ICU patients in Sub-Saharan Africa, including Ethiopia, have primarily focused on admission patterns, mortality rates, and their determinants. However, there is still uncertainty in the body of evidence regarding the pattern of disease, the incidence of MV, and the factors influencing clinical outcomes among patients with MV. The objective of this multicenter prospective cohort was to investigate the disease pattern and factors influencing clinical outcomes in patients receiving mechanical ventilation in Southern Ethiopia.

## Methods

### Study design

This Multi-center prospective cohort study was conducted in Southern Ethiopia teaching referral hospital ICUs from October 2020 to November 2022. Three teaching and referral hospital ICUs were selected randomly from ten teaching hospitals in Southern Ethiopia, namely Hawassa university referral hospital (HURH), Dilla University referral hospital (DURH), and Wolaita Sodo referral hospital (WURH). The ICUs are providing a similar level of care with almost similar staff profiles, monitoring modalities, ICU infrastructure, medical supplies, and admission patterns.

### Study inclusion and exclusion criteria

All patients over the age of 12 who received mechanical ventilation at DURH, Wolaita Sodo University Hospital, and Hawassa University Specialized Hospital ICUs and stayed there for longer than 24 h of admission were included; whereas, patients receiving non-invasive oxygen supplementation, patients who were discharged, transferred to, or left against medical advice before 24 h of mechanical ventilation, patients who were admitted to ICU on re-admission, and patients without attendants were excluded.

### Study variables

The cumulative incidence of 28-day mortality, duration of a mechanical ventilator, incidence of complications, pattern of disease, and length of stay in ICU were dependent variables, while socio-demographic characteristics: Age, sex, height, weight, BMI; Admission characteristics: date of ICU admission, date of discharge, vital sign, diagnosis, admission category, time of admission, laboratory index, disease severity score; Mechanical ventilation parameters: date of initiation, initial mode, date of tracheostomy, and date of weaning were independent variables.

### Data collection

**Fig. 1:**
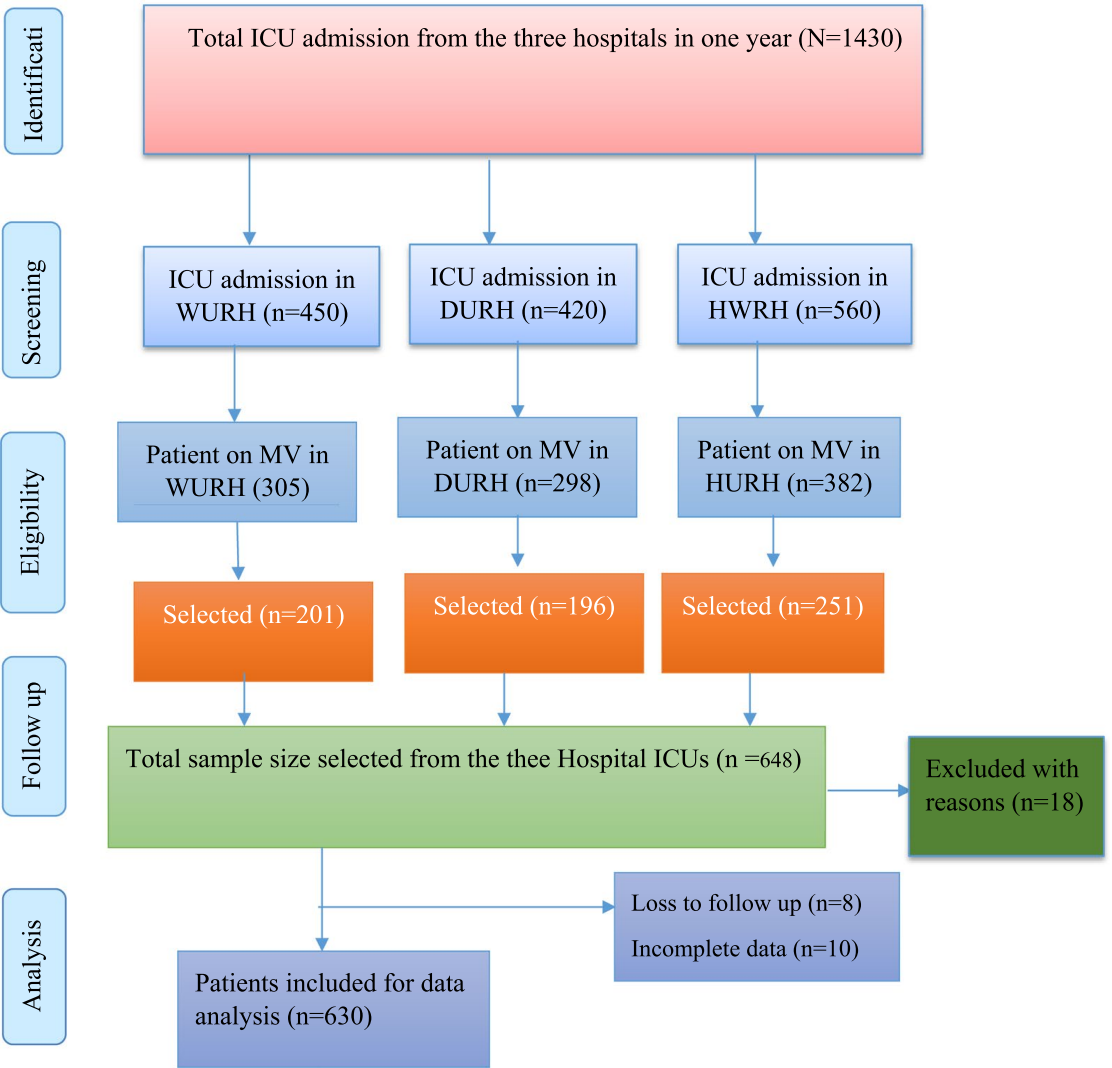
Strobe flow chart

The data were collected prospectively from three hospital ICUs patients in whom mechanical ventilation was initiated. After 2 h of mechanical ventilation, trained ICU nurses followed eligible participants for 28 days. The data were collected with a validated questionnaire and tools adopted from previous studies [11, 21–23]. A total of 630 patients were recruited consecutively from each ICU from October 2020 to November 2022 as per proportion allocation to size (Fig. 1).

The data collection included Socio-demographic characteristics: Age, Sex, Height, Weight, BMI); Admission characteristics: date of ICU admission, date of discharge, vital signs, diagnosis, admission category, time of admission, laboratory index, disease severity score; Complications: nosocomial infection, ventilator-associated pneumonia, sepsis, ARDS, aspiration, unplanned extubation, endotracheal tube blockage, tracheostomy loose/stenosis/fistula, cardiac arrest, acute kidney injury, bedsore); comorbidities: hypertension, diabetes mellitus, cardiovascular disease, Asthma/COPD, liver failure, renal failure, others, and clinical outcomes including mortality, duration of a mechanical ventilator, and complications were measured for 28 days. Furthermore, the disease severity scores including sequential organ assessment (SOFA) score, modified SOFA score, Acute Physiology, and Chronic Health Evaluation (APACHE) II score, and modified APACHE II score were recorded at admission and thereafter till discharge every seven days to compare the predicting ability and 28-day mortality of these tools.

### Statistical analysis

The data were checked, coded, and entered into Epi-info version 7.0 and imported to SPSS version 22 for analysis. However, STATA version 14 was used to produce some graphs, multilevel analysis and pool the incidence of clinical outcomes. Descriptive statistics were summarized with tables and figures. Categorical variables were reported in frequency and percentage. The numerical data were reported in mean ± SD for symmetric and median (Interquartile range) for asymmetric numeric data. The outlier of the data was checked with standardized residuals while Shapiro–Wilk tests were employed for the normality test. The multicollinearity among independent variables was checked by the Variance inflation factor (VIF), and a VIF of greater than 10 was considered multicollinearity. The association of demographic characteristics, admission category, indications for MV, and complications of MV with mortality were analyzed using multilevel binary logistic regression as there was clustering as depicted with an intraclass correlation coefficient (ICC = 8.5%). All variables showing significance on multilevel bivariate analysis at a p-value less than 0.25 will be taken to multilevel multivariate analysis. In multivariate analysis, a p-value of less than 0.05 was considered for the statistical association. This study was conducted in compliance with Strengthening the Reporting of Observational Studies in Epidemiology (STROBE) guidelines for observational studies [24].

### Study endpoints

The primary endpoints were the incidence of 28-day mortality and duration of mechanical ventilation, while the incidence of complications during MV was the secondary clinical outcome.

Definition incidence of clinical outcomes refers to the occurrence of new clinical events including mortality, morbidity, and prolonged mechanical ventilation in 28 days of ICU stay. Morbidities are defined as any complication developed during Mechanical ventilation which includes nosocomial infection, ventilator-associated pneumonia, sepsis, ARDS, aspiration, unplanned extubation, endotracheal tube blockage, tracheostomy loose/stenosis/fistula, cardiac arrest, acute kidney injury, and bedsore. Prolonged mechanical ventilation refers to the duration of mechanical ventilation for over 21 days [25, 26].

### Model building and comparison

The hierarchical nature of the hospital ICU care in which patients are nested within clusters, a multivariable multilevel logistic regression analysis was employed to account for this clustering effect. Thence, four models containing variables of interest were fitted for this study as follows: Model I (Null model) was fitted without explanatory variables to test random variability in the intercept and to estimate the intraclass correlation coefficient (ICC), Model II assessed the effects of individual-level predictors, Model III assessed the effects of hospital-level predictors, and Model IV (Full model) examined the effects of both individual and hospital-level characteristics simultaneously. The Akaike’s Information Criterion (AIC) and Bayesian Information Criterion (BIC) were used to select the model, and the model with low AIC and BIC values was considered the best-fitted model. Based on AIC and BIC, the full (model with individual and hospital-related variables) model had the smallest AIC and BIC value. Therefore, the full model best fits the data.

### Ethical statement

This study was reviewed and approved by Dilla University, College of Health Science and Medicine Research Ethics and Review Board (RERB), and a Unique Identification Number (UIN- duirb/001/21-08) was received. Besides, the protocol was registered retrospectively in ClinicalTrials.gov (NCT05303831) on March 30, 2022. The study was conducted in compliance with the Helsinki declaration for human and animal studies [27]. Informed consent was received from all participants’ legal attendants or guardians, and all patient identifiers were kept anonymous at all times. Besides, a formal letter was written to each University hospital ICU director to get permission.

## Results

### Sociodemographic and baseline characteristics

Out of 648 eligible cohorts, 630 (97.25) completed the 28-day prospective follow-up. The mean (standard deviation) age of the participants was 38.9 ± 17.6 years, where more than two-thirds of them were in the age ranges of 19 to 29 and > 40 years. The majority of participants were male, 426 (67.7%) (Table 1).

**Table 1:** Sociodemographic and Baseline Characteristics of Participants on Mechanical Ventilator in Southern Ethiopia Comprehensive Referral Hospitals (N = 630)

Variables	All patients (N = 630)	Survivors (N = 327)	Non-survivors (N = 303)	*P* value
Age (Mean ± SD)	38.9 ± 17.6	37.7 ± 6.1	40.2 ± 6.3	
< 18	47 (7.5)	28 (8.6)	19 (6.3)	< 0.001
19–29	231 (36.7)	120 (36.7)	111 (36.6)	
29–39	156 (24.8)	59 (18)	97 (32)	
> 40	196 (31)	120 (36.7)	76 (25.1)	
Sex				
Male	426 (67.6)	228 (69.2)	198 (65.3)	0.13
Female	204 (32.4)	99 (30.8)	105 (34.7)	
BMI	22.7 ± 1.7	22.7 ± 4.8	22.6 ± 0.6	0.001
*Respiratory Rate*				
< 35	507 (85.7)	305 (93.2)	202 (66.7)	< 0.001
> 35	123 (14.3)	22 (6.8)	101 (33.3)	
*Oxygen saturation*				
< 80	506 (80.3)	36 (11)	88 (29)	< 0.001
> 80	124 (19.7)	291 (89)	215 (71)	
*Glasgow coma scale*				
< 8	292 (46.4)	50 (15.3)	242 (79.9)	< 0.001
9–12	138 (21.9)	108 (33)	30 (9.9)	
12–15	200 (31.7)	169 (51.7)	31 (10.2)	
SBP	111.3 ± 30.6	110.6 ± 10.5	112.1 ± 2.1	< 0.001
DBP	69.7 ± 19.4	68.3 ± 7	71.2 ± 7.1	< 0.001

*SBP* Systolic blood pressure; *DBP* Diastolic blood pressure; *SD* Standard deviation; *BMI* Body Mass Index

### Diagnosis and indications for MV

This study showed that acute respiratory syndrome (ARDS) was the commonest diagnosis 178 (28.3%), followed by postoperative events 113 (17.9%), traumatic brain injury (TBI) 92 (14.6%), and stroke 80 (12.7%). The main reason for invasive mechanical ventilation was found to be respiratory failure 393 (62.4%) followed by airway protection 95 (15.1), and postoperative respiratory support 89 (14.1) (Additional file 2: Table S1).

### Admission characteristics

Of the 630 patients, medical cases accounted for more than half 356 (56.2) followed by trauma 125 (19.8%), and surgical cases, 89 (14.1%). The majority of patients were admitted to the ICU during day time and nighttime. Overall, 496 (78.7%) of the patients had comorbidities, where hypertension was the highest 151 (245) followed by respiratory (Asthma and COPD) 122 (19.4%), and DM 72 (11.4%). However, in a few patients, 42 (6.7%) had more than one comorbidity, while only 134 (21.2%) had unconfirmed comorbidities (Table 2).

**Table 2:** Admission characteristics of participants on mechanical ventilator in Southern Ethiopia Comprehensive Referral Hospitals (N = 630)

Variables	All patients (N = 630)	Survivors (N = 327)	Non-survivors (N = 303)	*P* value
*Patterns of admission*				
Medical	354 (56.2)	161 (49.2)	193 (63.7)	< 0.001
Surgical	89 (14.1)	68 (20.8)	30 (9.9)	
Trauma	125 (19.8)	72 (22)	53 (17.5)	
Others	53 (8.4)	26 (8)	27 (8.9)	
*Time of admission*				
Day time	339 (53.8)	190 (58.1)	149 (49.2)	0.001
Night	190 (30.2)	101 (30.9)	89 (29.4)	
Weekend	101 (16)	36 (11)	65 (21.4)	
*Comorbidity*				
Hypertension	151 (24)	57 (17.4)	94 (31.0)	< 0.001
DM	72 (11.4)	42 (12.8)	30 (9.9)	
CVS	60 (9.5)	46 (14.1)	14 (4.6)	
Asthma/COPD/TB	122 (19.4)	64 (19.6)	58 (19.1)	
More than one	42 (6.7)	23 (7.0)	19 (6.3)	
Others	49 (7.8)	16 (4.9)	33 (10.9)	
Unknown	134 (21.2)	79 (24.2)	55 (18.2)	
*Complications*				
VAP	198 (31.4)	114 (34.9)	84 (27.7)	< 0.001
Cardiac arrest	62 (9.8)	19 (5.8)	43 (14.2)	
Shock	54 (8.6)	22 (6.7)	32 (10.6)	
AKI	81 (12.9)	31 (9.5)	50 (16.5)	
Others	61 (9.7)	28 (8.6)	33 (10.9)	
None	174 (27.6)	113 (34.5)	61 (20.0)	
*Severity score*				
APACHE II	26.5 ± 18.5	15.2 ± 3.9	38.7 ± 6.2	< 0.001
MAPACHE II	24.8 ± 18.9	13.1 ± 3.6	37.5 ± 6.1	
SOFA	12.0 ± 5.8	8.4 ± 2.9	15.9 ± 4.0	
MSOFA	11.9 ± 5.7	8.2 ± 2.9	15.9 ± 4.0	
ALBUMIN	3.8 ± 1.8	3.8 ± 1.9	3.8 ± 2.0	
*Duration of MV*				
< 21 days	467 (74.1)	258 (78.9)	209 (69)	0.003
> 21 days	163 (25.9)	69 (21.1)	94 (31)	
*Mode of ventilation*				
VAC	386 (61.3)	149 (45.6)	237 (78.2)	< 0.001
PCV	88 (14)	66 (20.2)	22 (7.3)	
SIMV	116 (18.4)	83 (25.5)	33 (10.9)	
CPAP	40 (6.3)	29 (8.7)	11 (3.6)	

*CPAP* Continuous positive airway pressure; *SIMV* synchronized intermittent mandatory ventilation; *PCV* Pressure controlled ventilation; *VAC* Volume-assisted ventilation; *AKI* Acute kidney injury; *VAP* Ventilator associated pneumonia; *CVS:* cardiovascular system; *APACHE* Acute physiologic and chronic health evaluation; *MAPACHE* Modified acute physiologic and chronic health evaluation; *SOFA* Sequential organ failure assessment: *MSOFA* Modified sequential organ failure assessment

### Complications

The majority of patients 456 (71.7%) sustained at least one complication which included ventilator-associated pneumonia (VAP), cardiac arrest, shock, acute kidney injury (AKI), and others, where VAP alone accounted for 198 (31.4%) of the complications. The disease severity scores including Acute Physiologic and Health Evaluation II (APACHE II), Modified Acute Physiologic and Health Evaluation II (APACHE II), Sequential Organ Failure Assessment (SOFA), and Modified Sequential Organ Failure Assessment (MSOFA) were employed during admission of patients, and MAPACHE II) showed a significant association with 28-day survival status with strong predicting probability (Table 2).

### Incidence of clinical outcomes

**Fig. 2:**
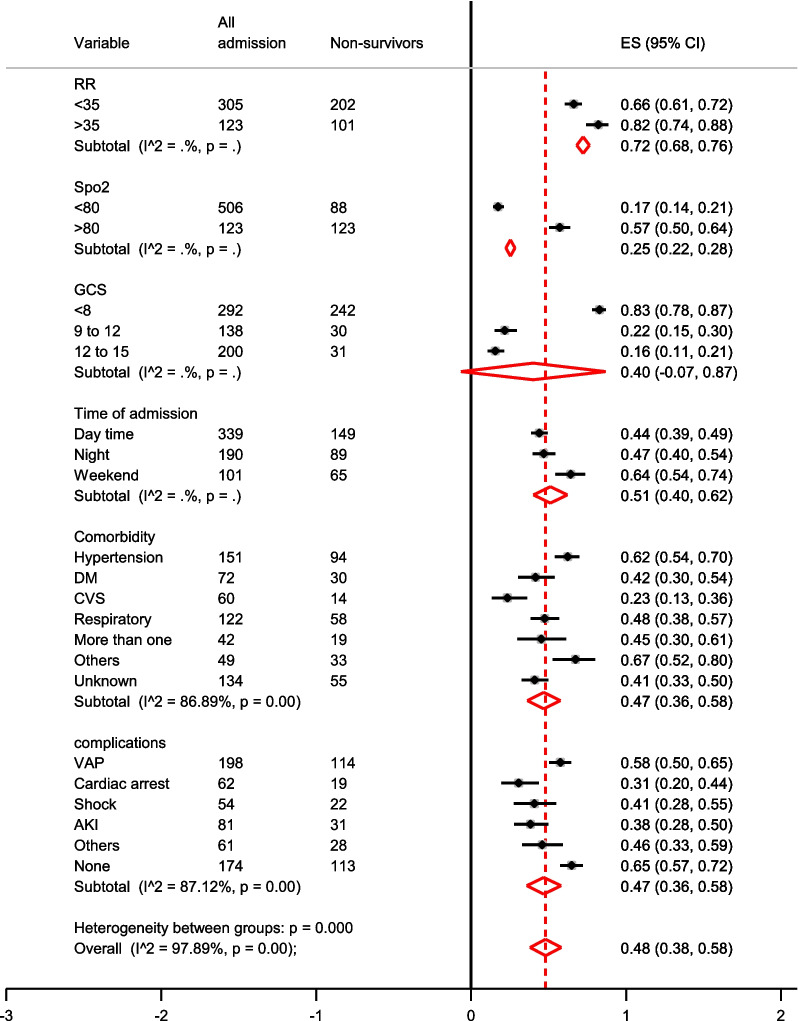
Forest plot for clinical parameters of Non-survivors among Intensive care Unit patients on mechanical ventilator; *AKI* Acute kidney injury; *VAP* Ventilator associated pneumonia; *CVS* Cardiovascular; *RR* Respiratory rate; *GCS* Glasgow coma scale; *DM* Diabetes

**Fig. 3:**
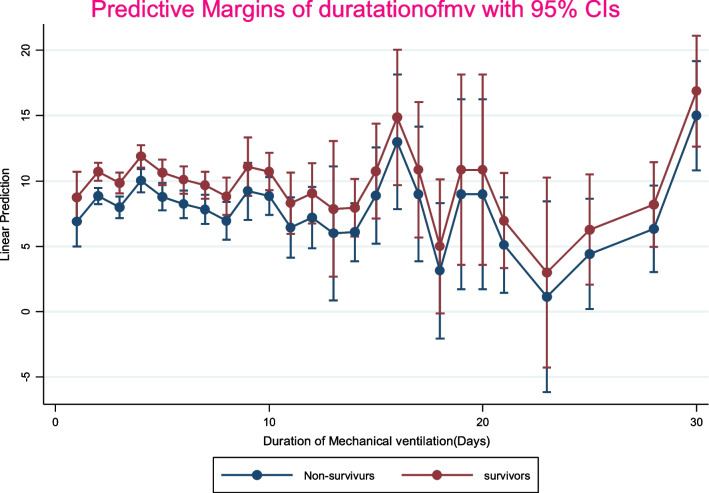
line graph with error bars showing duration of mechanical ventilation and survival status

**Fig. 4:**
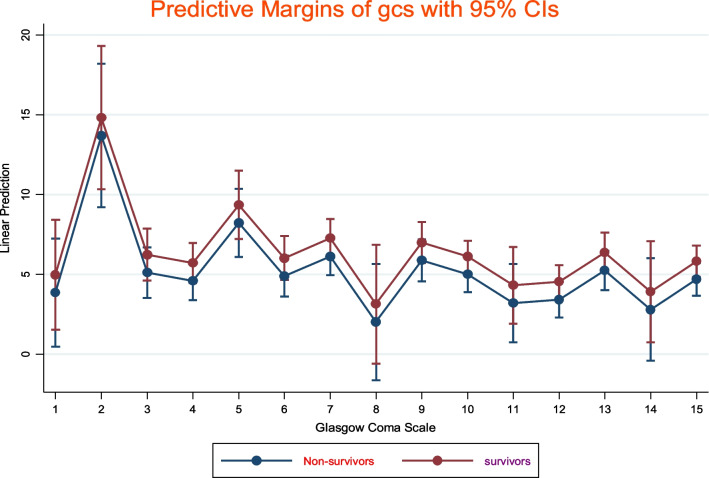
line graph with error bars showing effects of GCS on survival status among patients on mechanical ventilation in Southern Ethiopia, 2022

The incidence of 28-day mortality among ICU patients on a mechanical ventilator was 49% (95% CI: 36–58). The overall pooled prevalence of comorbidity among non-survivors was 47% (95% CI: 36–58) (Fig. 2). The histogram and normal distribution curve showed that the majority of patients were on mechanical ventilation for the first fifteen days (Additional file 1: Fig. S1). The duration of MV and baseline Glasgow Coma Scale did not show a significant difference in survival status as depicted on a line graph with error bars (Figs. 3 and 4).

### Mechanical ventilation variables

**Fig. 5:**
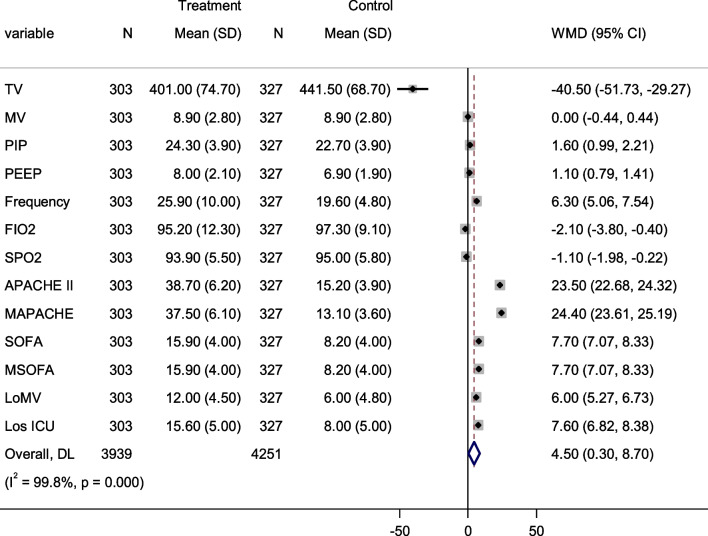
Forest plot for initial ventilator parameters, Deaths severity tools, length of mechanical ventilation among ICU patients on invasive mechanical ventilation: Treatment denotes to Non-survivors; control denotes survivors; *TV* Tidal volume; *MV* Minute ventilation; *PIP* Peak inspiratory pressure; *FI02* Fractional inspired oxygen; *SPo2* Percutaneous oxygen saturation; *APCHE* Acute physiologic and chronic health evaluation; *MAPACHE* Modified acute physiologic and chronic health evaluation; *SOFA* Sequential organ failure assessment; *MSOFA* Modified sequential organ failure assessment; *LoMV* Duration of MV; *Los ICU*: Length of stay in ICU

The mode of ventilation in the intensive care unit depends on the admission diagnosis, the severity of the disease, and the presence of other comorbidities. For example, patients with acute respiratory disease distress syndrome benefit from ArdsNet ventilator protocol which includes low tidal volume (4–8 ml/kg) predicted body weight, peak inspiratory pressure < 35 cmH20, and titrated FIO2 as per patient progress while patients with COPD require high peak flow rate with slow respiratory rate. This study tried to investigate the effects of ventilator parameters, duration of mechanical ventilation, and admission disease severity score on survival status and found a significant standardized mean difference in most of the parameters (Fig. 5).

### Prognostic scores

**Fig. 6:**
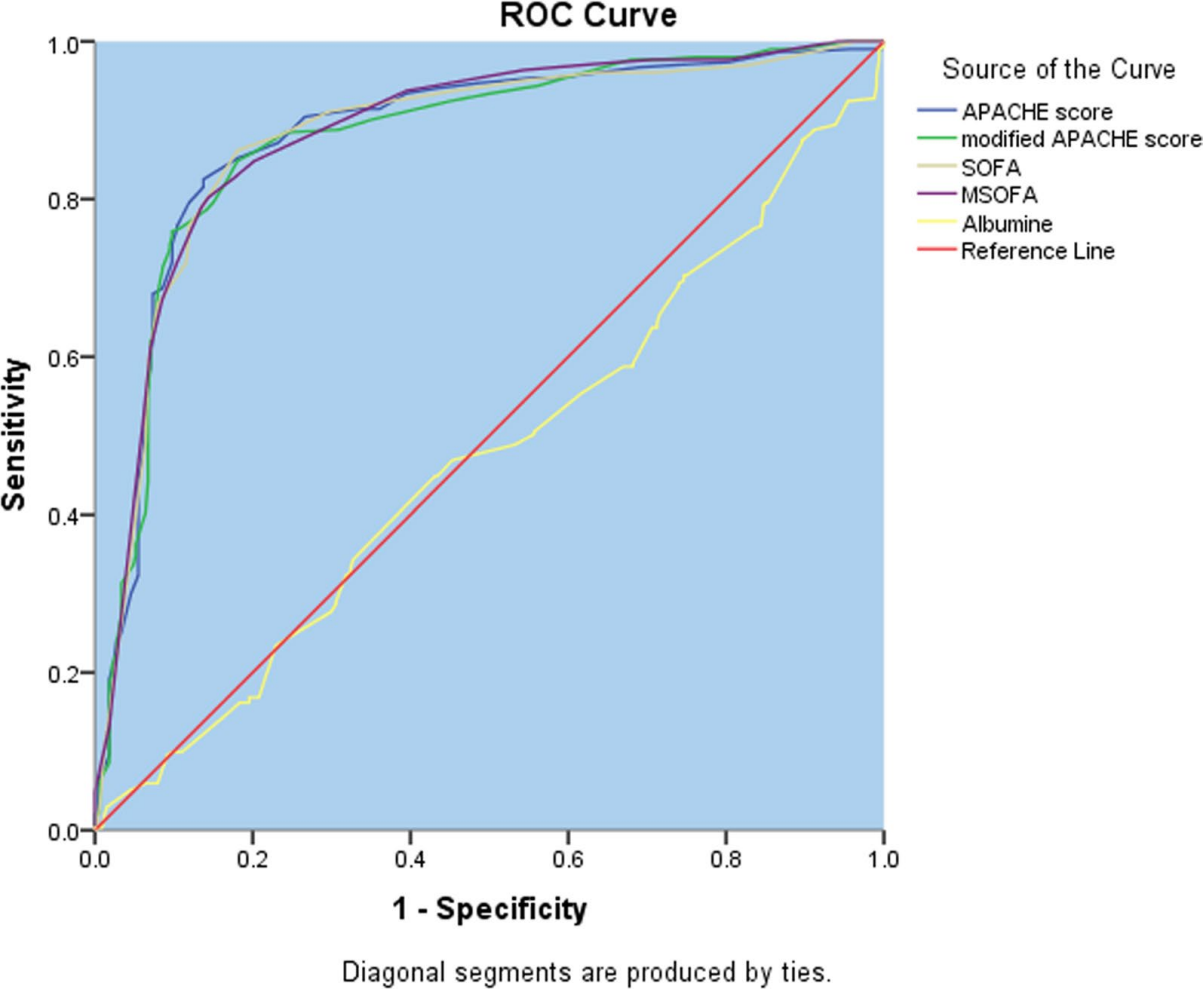
Receiver Operating Curve for the Prognostic Score APACHE II, Modified APACHE II, SOFA, and Modified SOFA

Several prognostic scores have been employed in the intensive care unit, which includes APACHE I-1 V, SOFA, and biomedical tests. However, the parameters used inquired are blood gas analysis and blood chemistry analysis which is not feasible in a resource-limited setting. In this regard, many studies have been conducted to compare the sensitivity, specificity, and predictability of modified tests that are applicable in a resource-limited environment, but the findings are yet inconsistent. This study was intended to compare the predictability of modified SOFA and modified APACHE II scores. The receiver operating curve showed the area under the curve for APACHE II, modified APACHE II, SOFA, and modified SOFA, revealing a similar prediction of 28-day mortality (Fig. 6 and Additional file 3: Table S2).

### Determinants of mortality

This multilevel multivariate analysis revealed that patients with medical causes of ICU admission were approximately 3 times (AOR = 2.9; 95% CI: 1.49–5.49, *P* value = 0.002) more likely to die compared to surgical (AOR = 1.3; 95% CI: 0.37–2.36), trauma (AOR = 0.9; 95% CI: 0.43–2.13), and other causes of admission (Additional file 4: Table S3 and 3).

**Table 3:** Multilevel multivariate analysis of the association between risk factors and 28-day mortality among ICU patients on Mechanical ventilators in Southern Ethiopia (N = 630)

Variables	All admission (N = 630)	Survival status		AOR (95% CI)	*P* value
		Yes	No		
Male	426 (67.6)	228 (69.2)	198 (65.3)	1.1 (0.69, 1.79)	0.668
Female	204 (32.4)	99 (30.8)	105 (34.7)	Reff	
*Age (yrs.)*					
≤ 81	47 (7.5)	28 (8.6)	19 (6.3)	Reff	
19 to 29	231 (36.7)	120 (36.7)	111 (36.6)	0.7 (0.29,1.67)	0.413
29 to 39	156 (24.8)	59 (18)	97 (32)	0.4 (0.16, 0.94)	0.037
≥ 40	196 (31)	120 (36.7)	76 (25.1)	0.9 (0.39, 2.28)	0.89
*Pattern of admission*					
Medical	354 (56.2)	161 (49.2)	193 (63.7)	2.9 (1.49, 5.49)	0.002
Surgical	89 (14.1)	68 (20.8)	30 (9.9)	1.3 (0.73, 2.36)	0.363
Trauma	125 (19.8)	72 (22)	53 (17.5)	0.9 (0.43, 2.13)	0.917
Others	53 (8.4)	26 (8)	27 (8.9)	Reff	
*Respiratory rate*					
< 35	507 (85.7)	305 (93.2)	202 (66.7)	Reff	
> 35	123 (14.3)	22 (6.8)	101 (33.3)	6.9 (3.87, 12.24)	< 0.001
*Comorbidities*					
Hypertension	151 (24)	57 (17.4)	94 (31.0)	2.1 (1.01, 4.42)	0.046
DM	72 (11.4)	42 (12.8)	30 (9.9)	7.4 (2.96, 18.38)	< 0.001
CVS	60 (9.5)	46 (14.1)	14 (4.6)	1.4 (0.78, 2.69)	0.244
More than one	42 (6.7)	23 (7.0)	19 (6.3)	3.9 (1.55, 9.89)	0.004
Others	49 (7.8)	16 (4.9)	33 (10.9)	1.4 (0.57, 3.67	0.045
Unknown	134 (21.2)	79 (24.2)	55 (18.2)	2.4 (1.21, 4.93)	0.011
Others	49 (7.8)	16 (4.9)	33 (10.9)	Reff	
*Time of ICU admission*					
Day time	339 (53.8)	190 (58.1)	149 (49.2)	2.0 (1.12, 3.53)	0.019
Night	190 (30.2)	101 (30.9)	89 (29.4)	2.5 (1.24, 5.05)	0.006
Weekend	101 (16)	36 (11)	65 (21.4)	Reff	
*Oxygen saturation*					
< 80	506 (80.3)	36 (11)	88 (29)	4.7 (2.69, 8.21)	< 0.001
> 80	124 (19.7)	291 (89)	215 (71)	Reff	
*Glasgow coma scale*					
< 8	292 (46.4)	50 (15.3)	242 (79.9)	5.9 (3.23, 10.69)	< 0.001
9–12	138 (21.9)	108 (33)	30 (9.9)	1.6 (0.98, 2.65)	0.061
12–15	200 (31.7)	169 (51.7)	31 (10.2)	Reff	

## Discussion

The discrepancies in sociodemographic, admission characteristics, disease severity, and presence of different comorbidities are considered strongly associated with patient clinical outcomes among patients in the intensive care unit. Therefore, this study has taken into consideration the potential clustering of variation in ICU service among patients on a mechanical ventilator in Southern Ethiopia.

Previous cross-sectional studies conducted in Ethiopia among intensive care patients focused on the overall pattern of admission, determinants, and clinical outcomes in the intensive care unit [13, 14, 19–21, 28, 29]. However, this multicenter prospective cohort study tried to investigate the pattern of admission, determinants, and clinical outcomes of patients on a mechanical ventilator.

This study showed that the main indication for initiation of mechanical ventilation was medical cases, followed by postoperative and trauma, which is in line with previous studies conducted in America [30], international cohort study [12], India [22], Poland [11], Ethiopia [13, 20, 21], and Brazil [31]. However, other studies conducted in Tanzania [32], Tunisia [33], the UK [34], and Ethiopia [19] showed that trauma was the main cause of the initiation of mechanical ventilation. These differences might be related to the study area where some ICUs might be specialized in admitting specific patients in the ICU while others admit mixed cases.

Regarding the comorbidity, hypertension was the main comorbidity followed by diabetes mellitus and respiratory disorders, which is consistent with an international cohort study including 16 countries [12], a study by Chiwhane and his colleagues in India [35], and a population-based study by Mehta et al. in the USA [30]. However, DM, cardiovascular, and COPD were the most prevalent comorbidities as per studies conducted by Karthikeyan et al. in India [22], and an international cohort study including 50 countries by Bellani et al., respectively [36]. This discrepancy may be related to the study area, a country where the older population with comorbidities is more prevalent.

The 28-day incidence of mortality in our study was 49% (95% CI: 36–58), which is very high when compared to previous studies conducted among ICU patients on a mechanical ventilator in middle and high-income countries [23, 34]. The disparity in mortality of patients requiring mechanical ventilation might be related to the delayed patient presentation, a lack of resources, insufficient healthcare infrastructure, inadequate trained medical staff, and financial constraints, which entail the stakeholders to buckle down to improve the patient’s clinical outcomes.

Although studies focusing specifically on the incidence of mortality requiring mechanical ventilation in developing countries are lacking, some authors reported cumulative mortality, where evidence from North and Sub-Saharan Africa showed inconsistent findings on the mortality of patients in ICUs receiving mechanical ventilation.

Contrary to the current study, the cumulative incidence of ICU mortality among patients with MV was the highest in Kenya (60.7%) [37], Tanzania (58.7%) [38], Ruanda (68.1%) [39], Tunisia (77.2%) [40], Nigeria (64%) [41], and Ethiopia (80%) [28]. However, other studies in the same Sub-Saharan African region showed a comparable and even lower incidence of mortality to the current study such as in Tanzania (41.4%) [42], Ethiopia (31.5%, 53.5%) [21, 43], and South Africa (24.4%) [44]. This discrepancy might be related to differences in the pattern of admission, sample size, ICU infrastructure, the inadequacy of medical supplies, lack of skilled manpower, and type of hospital and ICU.

The multilevel multivariate analysis revealed that DM comorbidity, baseline respiratory rate > 35 breaths/min, percutaneous arterial oxygen saturation < 80%, and GCS of less than eight were strong independent predictors of 28-day mortality in the intensive care unit among patients on mechanical ventilation. Besides, medical causes of admission, more than one comorbidity, and time of ICU admission were also independent predictors of mortality.

The current study revealed that being diabetic, having GSC < 8 night time admission, and having more than one comorbidity (AOR = 7.4; 95% CI: 2.96–18.38), (AOR = 5.9; (5% CI: 3.23, 10.69), (AOR = 2.5; 95%CI: 1.24, 5.05), and (AOR = 3.9; 95% CI: 1.55, 9.89) were independent predictors of 28-day mortality in patients receiving mechanical ventilation, respectively, which is consistent with previous studies [11, 23, 28, 34]. This calls for special attention to this kind of patient concerning nurse-to-patient ratio, availability of monitoring medication, skilled medical staff, portable diagnostic modalities, and nutritional supplementation.

### Strength and limitations

This is the first-ever multicenter prospective cohort study investigating mortality and determinants among patients on mechanical ventilation in sub-Saharan Africa. Besides, the study has taken into account the clustering effect of service variation by conducting a multilevel analysis. However, the study has certain limitations including a lack of arterial and blood chemistry tests to calculate disease severity scores in some patients, and the heterogeneity of cases as the ICUs were general hospitals that admitted a mix of cases.

### Implications for practice

This study revealed that the mortality of patients receiving mechanical ventilation in ICU was very high compared to previous studies conducted in western countries, which is strongly associated with the indication for mechanical ventilation, time of admission, baseline vital signs at admission, presence of comorbidities, and complications of mechanical ventilation. This entails mitigating strategies including but not limited to the provision of skilled medical staff, adequate medical supplies, adequate monitoring, and portable diagnostic modalities.

### Implications for further study

This is a multicenter prospective cohort study which is the first of its kind in Sub-Saharan Africa with multilevel analysis, but further multicenter prospective studies with a long period of follow-up might be inquired in specialized ICU setups of the homogenous population to investigate the certain impacts of the prolonged mechanical ventilator on patient outcomes.

## Conclusion

The higher 28-day mortality among ICU patients on mechanical ventilation might be attributed to factors such as delayed patient presentation, lack of resources, insufficient healthcare infrastructure, lack of trained staff, and financial constraints. The findings of this study could aid in the future improvement in healthcare services among ICU patients with MV in the region.

## Supplementary Information

**Additional file 1**. **Figure S3.** Duration of mechanical ventilation among patients in the intensive care Unit.

**Additional file 2**. **Supplemental Table S1:** Admission diagnosis and indication of Participants on Mechanical Ventilation in Southern Ethiopia Comprehensive Referral Hospitals (N = 630).

**Additional file 3**. **Supplemental Table S2:** Multilevel bivariate analysis of association between risk factors and 28-day mortality among ICU patients on Mechanical ventilator in Southern Ethiopia (N = 630).

**Additional file 4**. **Supplemental Table S3.** Area Under the curve for APACHE II, modified APACHE II, SOFA, modified SOFA, and albumin.

## Data Availability

Data and materials can be available where appropriate.
