# Supplementary material for: Pattern of disease and determinants of mortality among ICU patients on mechanical ventilator in Sub-Saharan Africa: a multilevel analysis
Source: Crit Care. 2023 Jan 24;27:37. doi: 10.1186/s13054-023-04316-w (PMC9875485; doi:10.1186/s13054-023-04316-w)
Supplement: Supplementary file 1 — Additional file 1. Figure S3. Duration of mechanical ventilation among patients in the intensive care Unit. [file 13054_2023_4316_MOESM1_ESM.docx]

**Figure S3: Duration of mechanical ventilation among patients in the intensive care Unit**
