# Supplementary material for: Pattern of disease and determinants of mortality among ICU patients on mechanical ventilator in Sub-Saharan Africa: a multilevel analysis
Source: Crit Care. 2023 Jan 24;27:37. doi: 10.1186/s13054-023-04316-w (PMC9875485; doi:10.1186/s13054-023-04316-w)
Supplement: Supplementary file 2 — Additional file 2. Supplemental Table S1: Admission diagnosis and indication of Participants on Mechanical Ventilation in Southern Ethiopia Comprehensive Referral Hospitals (N = 630). [file 13054_2023_4316_MOESM2_ESM.docx]

**Supplemental Table S1: admission diagnosis and indication of Participants on Mechanical Ventilation in Southern Ethiopia Comprehensive Referral Hospitals (N=630).**

| Variables | All patients(N=630) | Survivors(N=327) | Non-survivors (N=303) | P value |
| --- | --- | --- | --- | --- |
| Admission diagnosis | | | | |
| ARDS | 178(28.3) | 45(13.8) | 133(43.9) | <0.0001 |
| CAP | 35(5.6) | 15(4.6) | 20(6.6) |  |
| GBS | 5(0.8) | 4(1.2) | 1(0.3) |  |
| Septic shock | 22(3.5) | 6(1.8) | 16(5.3) |  |
| Stroke | 80(12.7) | 50(15.3) | 30(9.9) |  |
| Meningitis | 6(0.9) | 4(1.2) | 2(0.7) |  |
| Tetanus | 6(0.9) | 5(1.5) | 1(0.3) |  |
| Heart failure | 15(2.2) | 6(1.8) | 9(3) |  |
| TBI | 92(14.6) | 45(13.8) | 47(15.5) |  |
| Postoperative | 113(17.9) | 94(28.7) | 19(6.3) |  |
| Eclampsia | 13(2.3) | 8(2.5) | 5(1.6) |  |
| Others | 65(10.3) | 45(13.8) | 20(6.6) |  |
| Indication for Mechanical Ventilation | | | | |
| Respiratory Failure | 393(62.4) | 179(54.7) | 214(70.6) | <0.001 |
| Airway protection | 95(15.1) | 58(17.8) | 37(12.2) |  |
| Postoperative | 89(14.1) | 64(19.6) | 25(8.3) |  |
| Others | 53(8.4) | 26(7.9) | 27(8.9) |  |

ARDS: Acute Respiratory Distress Syndrome; CAP: Community Acquired Pneumonia; GBS: Gillian Barre Syndrome; TBI: Tuberculosis;
