# Supplementary material for: Pattern of disease and determinants of mortality among ICU patients on mechanical ventilator in Sub-Saharan Africa: a multilevel analysis
Source: Crit Care. 2023 Jan 24;27:37. doi: 10.1186/s13054-023-04316-w (PMC9875485; doi:10.1186/s13054-023-04316-w)
Supplement: Supplementary file 3 — Additional file 3. Supplemental Table S2: Multilevel bivariate analysis of association between risk factors and 28-day mortality among ICU patients on Mechanical ventilator in Southern Ethiopia (N = 630). [file 13054_2023_4316_MOESM3_ESM.docx]

**Supplemental Table S2:** multilevel bivariate analysis of association between risk factors and 28-day mortality among ICU patients on Mechanical ventilator in Southern Ethiopia (N=630)

| Variables | All admission(N=360) | Survival status | | COR(95% CI) | P value |
| --- | --- | --- | --- | --- | --- |
|  |  | Yes | No |  |  |
| Male | 426(67.6) | 228(69.2) | 198(65.3) | 1.3(0.89, 1.78) | 0.200 |
| Female | 204(32.4) | 99(30.8) | 105(34.7) | Reff |  |
| Age(yrs.) | | | | | |
| ≤18 | 47(7.5) | 28(8.6) | 19(6.3) | Reff |  |
| 19 to 29 | 231(36.7) | 120(36.7) | 111(36.6) | 0.7(0.34, 1.30) | 0.235 |
| 29 to 39 | 156(24.8) | 59(18) | 97(32) | 0.4(0.18, 0.72) | 0.004 |
| ≥40 | 196(31) | 120(36.7) | 76(25.1) | 0.9(0.5, 1.92) | 0.949 |
| Pattern of admission | | | | |  |
| Medical | 354(56.2) | 161(49.2) | 193(63.7) | 0.9(0.54, 1.78) | 0.95 |
| Surgical | 89(14.1) | 68(20.8) | 30(9.9) | 2.4(1.18, 4.89) | 0.015 |
| Trauma | 125(19.8) | 72(22) | 53(17.5) | 1.4(.71, 2.70) | 0.335 |
| Others | 53(8.4) | 26(8) | 27(8.9) | Reff | Reff |
| Respiratory Rate | | | | |  |
| <35 | 507(85.7) | 305(93.2) | 202(66.7) | Reff | Reff |
| >35 | 123(14.3) | 22(6.8) | 101(33.3) | 4.6(2.83, 7.32) | <0.001 |
| Comorbidities | | | | |  |
| Hypertension | 151(24) | 57(17.4) | 94(31.0) | 0.81(0.39, 1.66) | >0.05 |
| DM | 72(11.4) | 42(12.8) | 30(9.9) | 2.1(1.96, 4.71) | <0.001 |
| CVS | 60(9.5) | 46(14.1) | 14(4.6) | 4.4(1.86, 10.78) | <0.001 |
| More than one | 42(6.7) | 23(7.0) | 19(6.3) | 1.7(0.80, 3.43 | >0.05 |
| Others | 49(7.8) | 16(4.9) | 33(10.9) | 2.4(1.99, 5.87) | <0.001 |
| Unknown | 134(21.2) | 79(24.2) | 55(18.2) | 2.2(1.01, 4.48) | 0.005 |
| Others | 49(7.8) | 16(4.9) | 33(10.9) | Reff | Reff |
| Time of ICU admission | | | | |  |
| Day time | 339(53.8) | 190(58.1) | 149(49.2) | 2.2(1.38, 3.57) | 0.001 |
| Night | 190(30.2) | 101(30.9) | 89(29.4) | 2.7(1.59, 4.54) | <0.001 |
| Weekend | 101(16) | 36(11) | 65(21.4) | Reff | Reff |
| Oxygen saturation | | | | |  |
| <80 | 506(80.3) | 36(11) | 88(29) | 4.6(2.83, 7.32) | <0.001 |
| >80 | 124(19.7) | 291(89) | 215(71) | Reff | Reff |
| Glasgow Coma scale | | | |  |  |
| <8 | 292(46.4) | 50(15.3) | 242(79.9) | 7.3(4.36, 12.08) | <0.001 |
| 9-12 | 138(21.9) | 108(33) | 30(9.9) | 1.7(1.17, 2.67) | 0.007 |
| 12-15 | 200(31.7) | 169(51.7) | 31(10.2) | Reff | Reff |
