# Supplementary material for: Pattern of disease and determinants of mortality among ICU patients on mechanical ventilator in Sub-Saharan Africa: a multilevel analysis
Source: Crit Care. 2023 Jan 24;27:37. doi: 10.1186/s13054-023-04316-w (PMC9875485; doi:10.1186/s13054-023-04316-w)
Supplement: Supplementary file 4 — Additional file 4. Supplemental Table S3. Area Under the curve for APACHE II, modified APACHE II, SOFA, modified SOFA, and albumin. [file 13054_2023_4316_MOESM4_ESM.docx]

# Supplemental Table S3 Area Under the curve for APACHE II, modified APACHE II, SOFA, modified SOFA, and albumin

| Test result variables | Area under the curve | Std. Error | Asymptomatic sig. | Asymptomatic 95% confidence interval | |
| --- | --- | --- | --- | --- | --- |
|  |  |  |  | Lower Bound | Upper Bound |
| APACHE score | .882 | .015 | .000 | .853 | .910 |
| MAPACHE | .877 | .015 | .000 | .848 | .905 |
| SOFA | .878 | .015 | .000 | .849 | .907 |
| MSOFA | .883 | .014 | .000 | .855 | .910 |
| Albumin | .472 | .023 | .224 | .427 | .517 |

APACHE: Acute physiologic and Chronic Health Evaluation; MAPACHE: Modified Acute physiologic and Chronic Health Evaluation; SOFA: Sequential Organ Failure Assessment; MSOFA: Modified Sequential Organ Failure Assessment
